# Putative New Lineage of West Nile Virus, Spain

**DOI:** 10.3201/eid1603.091033

**Published:** 2010-03

**Authors:** Ana Vázquez, María Paz Sánchez-Seco, Santiago Ruiz, Francisca Molero, Lourdes Hernández, Juana Moreno, Antonio Magallanes, Concepción Gómez Tejedor, Antonio Tenorio

**Affiliations:** Centro Nacional de Microbiología–Instituto de Salud Carlos III, Majadahonda, Spain (A. Vázquez, M.P. Sánchez-Seco, F. Molero, L. Hernández, A. Tenorio); Diputación Provincial de Huelva, Huelva, Spain (S. Ruiz, J. Moreno, A. Magallanes); Laboratorio Central de Veterinaria, Algete, Spain (C. Gómez Tejedor)

**Keywords:** West Nile virus, Spain, flavivirus, putative new lineage, viruses, zoonoses, dispatch

## Abstract

To ascertain the presence of West Nile virus (WNV), we sampled mosquitoes in 2006 in locations in southern Spain where humans had been infected. WNV genomic RNA was detected in 1 pool from unfed female *Culex pipiens* mosquitoes. Phylogenetic analysis demonstrated that this sequence cannot be assigned to previously described lineages of WNV.

West Nile virus (WNV) has been described in Africa, Europe, the Middle East, Asia, Australia, and, most recently, the Americas. Over the last few years, many reports about WNV have been published after the outbreaks in Romania, Morocco, Italy, Russia, and Israel, but especially with the introduction and spread of the virus in the Americas. Currently, the virus has a wide geographic distribution, and WNV infection is considered an emerging zoonosis (*1*).

Although only WNV lineage 1 is present in the Americas, >5 lineages of the virus seem to circulate in the Old World (*2*). In 2008, several countries in Europe reported WNV activity due to different lineages. WNV lineage 1 was isolated from horses and birds in northern Italy, and WNV infection was described in 6 persons (*3*). The Austrian veterinary authorities reported 2 outbreaks of WNV in wild birds, 1 in northern Austria, and 1 in the region of Vienna. The virus isolated from these birds, sparrow hawks, was WNV lineage 2 and was very homologous to 2 strains previously found in goshawks in Hungary in 2004 and 2005. These reports represented the emergence of a WNV lineage 2 strain outside Africa for the first time (*4*). Migratory birds that overwintered in central Africa may have recently introduced this exotic strain in the wetlands of different eastern European countries. Consequently, this neurotropic, exotic WNV strain may become a resident pathogen in Europe with public health consequences.

A new lineage of WNV (named Rabensburg virus), of as yet unknown human pathogenicity, was isolated from *Culex pipiens* mosquitoes in 1997 and 1999 on the Czech Republic–Austria border, only a few hundred kilometers from the region where WNV emerged in Hungary (*5*). The Rabensburg isolate 97–103, obtained from *Cx. pipiens* mosquitoes (1997) in Czech Republic (*6*), and LEIVKrnd88–190, isolated from *Dermacentor marginatus* ticks in a valley in the northwestern Caucasus Mountains in 1998 (*7*), have been proposed to be novel variants of WNV. These isolates are genetically different from viruses of lineage 1 and 2 and have been proposed as members of lineages 3 and 4, respectively. Moreover, 2 other related viruses show no clear relationships with WNV, the strain KUN MP502–66 from Malaysia, and Koutango (KOUV), an African virus, with poor statistical support for clustering with either of the WNVs, which suggests that they represent 2 single-isolate lineages (*8*).

Previous serologic surveys conducted with small rodents and humans in different areas of Spain have shown evidence of WNV circulation (*9*). Although no neurologic illness outbreaks have been documented in Spain, recent studies indicate that WNV is circulating in the southern part of the country, close to the areas of the recent foci in Portugal and Morocco. This part of Spain contains several wetlands, which have high densities of migratory birds and mosquitoes. WNV activity has been reported in this region on the basis of serologic surveys in birds, horses, and humans (*10*–*12*). Moreover, the first clinical case of WNV infection in Spain was reported in 2004 in a patient visiting southwestern Spain (*13*), and WNV lineage 1 was detected and further isolated in free-living and captive Spanish golden eagles in south-central Spain (*14*). Following up these results, we collected mosquito samples especially from areas from which positive serum samples had been obtained to look for WNV in its vector.

## The Study

The area of study included 2 wetlands: Marismas del Odiel (tidal marshes) and Doñana (freshwater marshes), both located in southwestern Spain. Mosquitoes were captured in 2006 with U.S. Centers for Disease Control and Prevention light traps supplied with CO_2_ and with gravid traps, which were used in the field during the late afternoon and retrieved the following morning. Mosquitoes were pooled by species, sex, collecting site, and date. The number of mosquitoes per pool ranged from 1 to 50. Mosquitoes/pools were homogenized in a range of 500–700 μL of minimal essential medium supplemented with 200 U/mL of antimicrobial drugs (penicillin/streptomycin) and 10% of fetal bovine serum and then were stored at –80°C until they were tested for flavivirus. The homogenate was centrifuged at 13,000 rpm for 5 min at 4°C, and the screening was performed with a generic nested reverse transcription PCR (*15*) to detect flavivirus genome.

This study comprised 35,424 mosquito specimens grouped in 1,641 pools and representing 14 species (Table 1). Approximately 11% of the pools (191) showed a positive result for flavivirus amplification. However, WNV was identified in only 1 (pool HU2925/06). The pool contained 50 unfed *Cx. pipiens* complex females, captured in June 2006 in Palos de la Frontera (Huelva; latitude 37°12′41.76′′N; longitude 6°55′9.58′′W).

**Table 1:** Mosquitoes collected and tested for flavivirus in Huelva, Spain, 2006

Species	No. pools	No. positive pools
*Anopheles algeriensis*	6	0
*An. atroparvus*	21	0
*An. claviger*	1	0
*Anopheles* sp.	1	0
*Coquillettidia richiardii*	2	0
*Ochlerotatus caspius*	491	33
*Oc. detritus*	71	1
*Oc. geniculatus*	2	0
*Ochlerotatus sp.*	2	0
*Culex modestus*	131	1
*Cx. perexiguus*	54	0
*Cx. pipiens*	457	3
*Cx. theileri*	308	151
*Culex* sp.	28	2
*Culiseta annulata*	13	0
*Cs. longiareolata*	51	0
*Cs. subochrea*	1	0
*Culiseta* sp.	1	0

Total

1,641

191

A fragment of 1,813 nucleotides (nt) from the nonstructural protein 5 (NS5) gene from this WNV genome (GenBank accession no. GU047875) was amplified by using 3 WNV-specific nested-PCRs designed in this study. The phylogenetic analysis resulted in a tree in which, as expected, this sequence fell under the branch of WNV, with a value of certainty of 100% (Figure, panel A). A common evolutionary branch between the Spanish strain and lineage 4 (99% certainty) can be observed, and both strains seem related to lineage 3. Sequence differences observed between HU2925/06 and other strains of WNV are shown in Table 2. The minor genetic distance was obtained for lineage 4 and the highest for lineage 5. To confirm that the sequence detected in Spain did not correspond to those of the isolates KUN MP502–66 and KOUV, we analyzed part of the genome sequence of both viruses, and partial sequences showed that these viruses cluster into a distinct genetic lineage (Figure, panel B). The sequence data for KOUV was retrieved from GenBank (strain Koutango DakArD1470, accession no. AF013384), and the partial sequence for KUN MP502–66 was obtained in this work amplifying part of the NS5 gene (GenBank accession no. GU047874).

**Figure:**
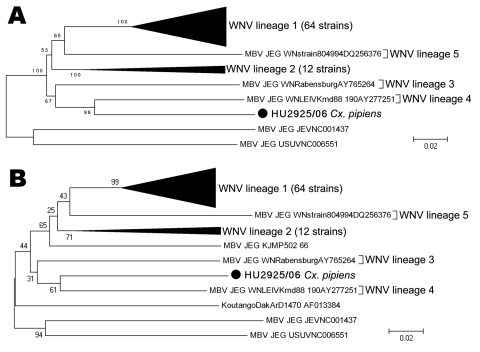
Phylogenetic tree of 79 WNV isolates by the neighbor-joining method and distance-p model on MEGA3.1 (www.megasoftware.net/mega_dos.html). Bootstrap values correspond to 1,000 replications. A) Analysis of a 1,813-nt fragment of the nonstructural protein 5 (NS5) gene. B) Analysis of the 800-nt fragment of the NS5 gene. KOUV (strain DakArD1470, AF013384) and Malaysia (strain KUN MP502–66, GU047874) (**boldface**) were also used to obtain this tree. Scale bars indicate nucleotide substitutions per site. WNV, West Nile virus; nt, nucleotide; MBV, mosquito-borne viruses; JEG, Japanese encephalitis group; JEV, Japanese encephalitis virus; USUV, Usutu virus. Viruses used in the phylogenetic study (GenBank accession nos.): WNV lineage 1 (AY712948, AY712947, AY490240, AY278442, AY278441, AY277252, AF404757, AF404756, AF404755, AF404754, AF404753, AF481864, AY603654, AY646354, AY289214, AY795965, AY842931, AY660002, AF196835, DQ164206, DQ164202, DQ164197, AF260969, AF260968, AF260967, DQ211652, DQ164204, DQ164200, DQ164201, AF533540, DQ005530, DQ118127, DQ164205, DQ164203, DQ164199, D00246, DQ164196, DQ080058, DQ080054, DQ080055, DQ080056, DQ164193, DQ164186, DQ164195, DQ164191, DQ164205, DQ080053, AY848696, AB185917, DQ080052, AB185914, DQ080051, DQ164189, AF404756, DQ164190, AY712945, AY712946, DQ080059, DQ164188, DQ164187, DQ164192, AF404757, AY277252, AY274505); WNV lineage 2 (DQ116961, DQ318019, M12294, EF429200, AY532665, EF429198, EF429199, EF429197, NC001563, AY688948, DQ176636, DQ318020); WNV lineage 3 (AY765264); WNV lineage 4 (AY277251); WNV lineage 5 (DQ256376); Spanish WNV (HU2925/06, GU047875); JEV: (NC001437); and USUV (NC006551) (as an outgroup).

**Table 2:** Sequence differences between HU2925/06 and other strains representing previously described West Nile virus lineages or related flaviviruses*

Lineages	Nucleotide difference, %							Amino acid difference, %		
	1a	1b	2	3	4	5		HU2925/06	JEV	USUV
1a	–	1.1	5	6.7	7.8	6		8.3	17.8	16.5
1b	11.1	–	4.8	6.8	7.6	6		8.1	17.9	16.7
2	20	21.4	–	5.5	6.1	6.7		7.7	18.5	17.6
3	21.6	22	20.8	–	6.8	7.8		8.6	18.1	18.1
4	21.8	22	22	22.6	–	9.5		5	19.6	19.1
5	19.6	20.2	21.3	22.3	23.4	–		10.9	18.4	17.1
HU2925/06	22.5	22.4	22.2	22	18.3	23.4		–	19.1	19.2
JEV	27.3	28.2	27.1	26.9	29	27.7		28	–	14.3
USUV	26.1	26.8	27.8	29.1	28.1	27.6		28.2	25	–

*Values of nucleotide and amino acid differences were calculated by p distance and multiplied by 100. JEV, Japanese encephalitis virus; USUV, Usutu virus.

To isolate the virus, the positive pool was diluted 1:20 in the minimal essential medium, and 200 µL were injected onto C6/36 (*Aedes albopictus* cells), RK-13 (rabbit kidney cells), and Vero (African green monkey cells) monolayer cells grown at a constant temperature for each cell line (33°C, 37°C, and 37°C, respectively). Cell cultures were incubated under the same conditions for 7 days, and 3 blind passages were carried out. Signs of cytopathic effect were checked daily, and the culture supernatants were tested by reverse transcription–PCR. Neither cytopathic effect nor amplification was obtained. No virus was isolated from any of the 3 cell cultures.

## Conclusions

The phylogenetic analysis performed on a 1,813-nt fragment of the NS5 gene clearly shows that the sequence recovered in Spain grouped within the branch of WNV with high values of certainty (100%). The tree topology shows a common evolutionary branch between the Spanish WNV genome (HU2925/06) and lineage 4, which clusters close to lineage 3. The lineages 3 and 4 were detected recently in Europe (1997 and 1998, respectively), and they have not been previously associated with natural disease in vertebrates. In addition, the phylogenetic analysis performed on 800 nt fragments of the NS5 gene indicated that the Spanish strain was not the same that KUN MP502–66 and KOUV, and that KUN MP502–66 seems to be a different lineage.

This report and the recent description of WNV lineage 1 in wild birds (*14*) demonstrate the circulation of both WNV lineages in Spain. This finding should lead to the analysis of serologic evidence of WNV infections in birds, horses, and humans in Spain and surrounding countries, where the highly pathogenic WNV strains sporadically cause clinical infections. An explanation for the high WNV seroprevalence levels found in birds, horses, and humans in the absence of neurologic disease in Spain could be that this new lineage infects birds and protects them from most pathogenic strains of WNV.
